# Supplementary material for: Evidence-based public health messaging on the non-visual effects of ocular light exposure: a modified Delphi expert consensus
Source: BMJ Public Health. 2025 Oct 10;3(2):e003205. doi: 10.1136/bmjph-2025-003205 (PMC12519682; doi:10.1136/bmjph-2025-003205)
Supplement: online supplemental appendix 1 [file bmjph-3-2-s001.docx]

# Appendix A: Consortium members

## Coordinating committee

| **Name** | **Institution** | **Country** | **ORCID** |
| --- | --- | --- | --- |
| Prof. Manuel Spitschan | Technical University of Munich & Max Planck Institute for Biological Cybernetics | Germany | 0000-0002-8572-9268 |
| Dr Laura Kervezee | Leiden University Medical Center | Netherlands | 0000-0002-6062-9164 |
| Dr Oliver Stefani | Lucerne University of Applied Sciences and Arts | Switzerland | 0000-0003-0199-6500 |
| Dr Marijke Gordijn | Chrono@Work & University of Groningen | Netherlands | 0000-0001-9521-8085 |
| Dr Jennifer A. Veitch | National Research Council of Canada | Canada | 0000-0003-3183-4537 |
| Dr Renske Lok | Stanford University | USA | 0000-0003-1684-5625 |

## Light for Public Health Consortium (in alphabetical order):

| **Name** | **Institution** | **Country** | **ORCID** |
| --- | --- | --- | --- |
| Dr Christine Blume | University of Basel & Psychiatric Hospital of the University of Basel | Switzerland | 0000-0003-2328-9612 |
| Prof. George C. Brainard | Thomas Jefferson University | USA | 0000-0003-4472-5786 |
| Dr Kai Broszio | Federal Institute for Occupational Safety and Health (BauA) | Germany | 0000-0002-8269-8654 |
| Prof. Timothy Brown | University of Manchester | United Kingdom | 0000-0002-5625-4750 |
| Jan Denneman | Good Light Group | Netherlands | 0009-0006-4456-4301 |
| Dr Maydel Fernandez-Alonso | Max Planck Institute for Biological Cybernetics | Germany | 0000-0002-3179-7476 |
| Prof. Shigekazu Higuchi | Kyushu University | Japan | 0000-0001-7131-0792 |
| Dr Daniel S. Joyce | University of Southern Queensland | Australia | 0000-0002-6407-4653 |
| Prof. Robert Lucas | University of Manchester | United Kingdom | 0000-0002-1088-8029 |
| Dr Elise M. McGlashan | University of Melbourne | Australia | 0000-0002-3864-7198 |
| Dr Raymond P. Najjar | National University of Singapore & Singapore Eye Research Institute | Singapore | 0000-0002-3770-2300 |
| Dr Luke Price | UK Health Security Agency | United Kingdom | 0000-0002-4528-0566 |
| Dr Sylvia Rabstein | Ruhr University Bochum | Germany | 0000-0002-1757-847X |
| Dr Luc Schlangen | Eindhoven University of Technology | Netherlands | 0000-0002-8424-7240 |
| Dr David H. Sliney | IES Photobiology Committee | USA | 0000-0003-4859-5982 |
| Dr Juliëtte van Duijnhoven | Eindhoven University of Technology | Netherlands | 0000-0003-1145-7033 |
| Dr Daniela Weiskopf | Federal Office for Radiation Protection | Germany | 0009-0001-8637-7911 |
| Prof. Kenneth Wright | University of Colorado Boulder | USA | 0000-0002-1781-7469 |
| Dr Johannes Zauner | Technical University of Munich & Max Planck Institute for Biological Cybernetics | Germany | 0000-0003-2171-4566 |
